# Effect of continuous positive airway pressure treatment of obstructive sleep apnea-hypopnea in multiple sclerosis: A randomized, double-blind, placebo-controlled trial (SAMS-PAP study)

**DOI:** 10.1177/13524585211010390

**Published:** 2021-04-23

**Authors:** Sulaiman Khadadah, R John Kimoff, Pierre Duquette, Vincent Jobin, Yves Lapierre, Andrea Benedetti, Fatema T Johara, Ann Robinson, Elaine Roger, Amit Bar-Or, Gabriel Leonard, Marta Kaminska, Daria A Trojan

**Affiliations:** Respiratory Division and Sleep Laboratory, McGill University Health Centre, Montreal, QC, Canada; Respiratory Division and Sleep Laboratory, McGill University Health Centre, Montreal, QC, Canada; Department of Neuroscience, Centre Hospitalier de l’Université de Montréal, Université de Montréal, Montreal, QC, Canada; Pulmonary Division, Department of Medicine, Centre Hospitalier de l’Université de Montréal, Université de Montréal, Montreal, QC, Canada; Department of Neurology and Neurosurgery, Montreal Neurological Institute-Hospital, McGill University Health Centre, McGill University, Montreal, QC, Canada; Department of Epidemiology, Biostatistics and Occupational Health, McGill University, Montreal, QC, Canada; Department of Neurology and Neurosurgery, Montreal Neurological Institute-Hospital, McGill University Health Centre, McGill University, Montreal, QC, Canada; Department of Neurology and Neurosurgery, Montreal Neurological Institute-Hospital, McGill University Health Centre, McGill University, Montreal, QC, Canada; Department of Neuroscience, Centre Hospitalier de l’Université de Montréal, Université de Montréal, Montreal, QC, Canada; Center for Neuroinflammation and Experimental Therapeutics and Department of Neurology, University of Pennsylvania, Philadelphia, PA, USA; Department of Neurology and Neurosurgery, Montreal Neurological Institute-Hospital, McGill University Health Centre, McGill University, Montreal, QC, Canada; Respiratory Division and Respiratory Epidemiology and Clinical Research Unit, McGill University Health Centre, McGill University, Montreal, QC, Canada; Department of Neurology and Neurosurgery, Montreal Neurological Institute-Hospital, McGill University Health Centre, McGill University, Montreal, QC, Canada

**Keywords:** Multiple sclerosis, clinical trial, sleep apnea, obstructive, continuous positive airway pressure, fatigue, sleepiness, sleep

## Abstract

**Objective:**

The aim of this study was to evaluate the effect of continuous positive airway pressure (CPAP) treatment on the Fatigue Severity Scale (FSS, preplanned primary outcome), another fatigue measure, sleep quality, somnolence, pain, disability, and quality of life in multiple sclerosis (MS) patients with obstructive sleep apnea-hypopnea (OSAH).

**Methods:**

In a randomized, double-blind trial (NCT01746342), MS patients with fatigue, poor subjective sleep quality, and OSAH (apnea-hypopnea index of ⩾ 15 events per hour/sleep), but without severe OSAH (apnea-hypopnea index > 30, and 4% oxygen desaturation index > 15 events/hour or severe somnolence), were randomized to fixed CPAP or sham CPAP for 6 months. Outcome assessments were performed at 3 and 6 months.

**Results:**

Of 49 randomized patients, 34 completed the protocol. Among completers, FSS did not improve with CPAP compared to sham at 6 months. FSS tended to improve (*p* = 0.09), and sleepiness (Epworth Sleepiness Scale) improved significantly (*p* = 0.03) at 3 months with CPAP compared to sham, but there were no other improvements with CPAP at either study evaluation.

**Conclusion:**

In non-severe OSAH patients, CPAP did not significantly improve the primary outcome of FSS change at 6 months. In secondary analyses, we found a trend to improved FSS, and a significant reduction in somnolence with CPAP at 3 months.

## Introduction

Fatigue is the most common and typically most disabling symptom of multiple sclerosis (MS). In MS, sleep disturbances are common,^1,2^ clinically under-recognized and contribute to fatigue.^3–5^ Poor sleep quality, assessed using the Pittsburgh Sleep Quality Index (PSQI),^6^ was present in 62% of 504 MS patients versus 32% of normal controls.^1^ Sleep quality and objective polysomnographic parameters also correlate with quality of life in MS.^1,7^ There is a paucity of studies that have objectively evaluated sleep-disordered breathing in MS.

In a previous study, we evaluated sleep disorders using overnight in-laboratory polysomnography (PSG), and clinical symptoms and quality of life using standardized questionnaires in 62 MS patients. We found a high frequency of sleep disorders, most commonly obstructive sleep apnea-hypopnea (OSAH) in 58% of patients, and a significant association of fatigue with severe OSAH.^8^ Patients were offered treatment as necessary by a sleep physician. Of the 62 patients, 56 returned for a follow-up evaluation. In a non-randomized, controlled study, treatment of OSAH had a significant beneficial effect on fatigue, somnolence and sleep quality in adjusted, multivariate analyses.^9^ Other investigators have also found an association of OSAH with fatigue.^10–12^ In addition, non-randomized follow-up studies indicate that OSAH treatment may improve fatigue.^13,14^ Continuous positive airway pressure (CPAP) is the treatment of choice for OSAH in the general population, but the potential benefits of CPAP in MS remained to be evaluated in a randomized, controlled clinical trial.

We hypothesized that fatigued MS patients with OSAH who are treated with CPAP would experience an improvement in fatigue and other clinical parameters compared to a control group not treated with CPAP. The objectives of this study were to (1) determine the clinical effectiveness of a 6-month course of CPAP treatment for OSAH on the Fatigue Severity Scale (FSS; preplanned primary outcome)^15^ in MS patients with OSAH and (2) evaluate the effect of CPAP treatment on the secondary outcomes of another fatigue measure, sleep quality, somnolence, pain, disability, and quality of life in MS patients with OSAH at 3 and 6 months.

## Methods

### Study design

The study was a randomized, double-blinded, placebo-controlled trial conducted at the McGill University Health Centre (primary center) and the Centre Hospitalier de l’Université de Montréal from January, 2013 through October, 2018. The study was approved by our institutional research ethics boards (ID no. NEU-11-043 and 2013-3229, CE 12.099-BSP), and all patients provided written informed consent. This trial was registered at ClinicalTrials.gov (NCT01746342).

### Patients

We recruited patients from our MS Clinics with an established diagnosis of MS^16^ and Expanded Disability Status Scale (EDSS)^17^ score of 0 to 7, who were relapse-free for at least 30 days and stable on disease modifying treatment for 3 months prior to screening. Additional inclusion criteria were poor sleep quality by PSQI^6^ score > 5, substantial fatigue by FSS^15^ score ⩾ 4, no more than minimal cognitive impairment with a Montreal Cognitive Assessment (MoCA)^18^ score of ⩾ 26, and a diagnosis of OSAH by PSG with a total apnea-hypopnea index (AHI) ⩾ 15 events/hour of sleep. Exclusion criteria were pregnancy, psychiatric conditions which preclude informed consent or study requirements, or other significant neurological or active medical conditions, clinically significant laboratory abnormalities on screening blood tests, current OSAH treatment, or other symptomatic sleep disorder. Patients with severe OSAH (AHI > 30) with either a 4% oxygen desaturation index ⩾ 15 events/hour, work in a safety–critical position or severe sleepiness Epworth Sleepiness Scale Score (ESS)^19^ ⩾ 15 were excluded for ethical reasons.

### Randomization and blinding

Randomization (DACIMA software) was via a randomized block scheme with two stratification factors: study center and baseline fatigue level (FSS).

Participants, evaluating physicians, study coordinators, and statisticians were blinded to treatment assignment. However, the sleep physicians, sleep technologists, and respiratory therapists installing CPAP were not blinded. The latter were specially trained not to break the blind.

### Procedures/visits

**Figure 1.**
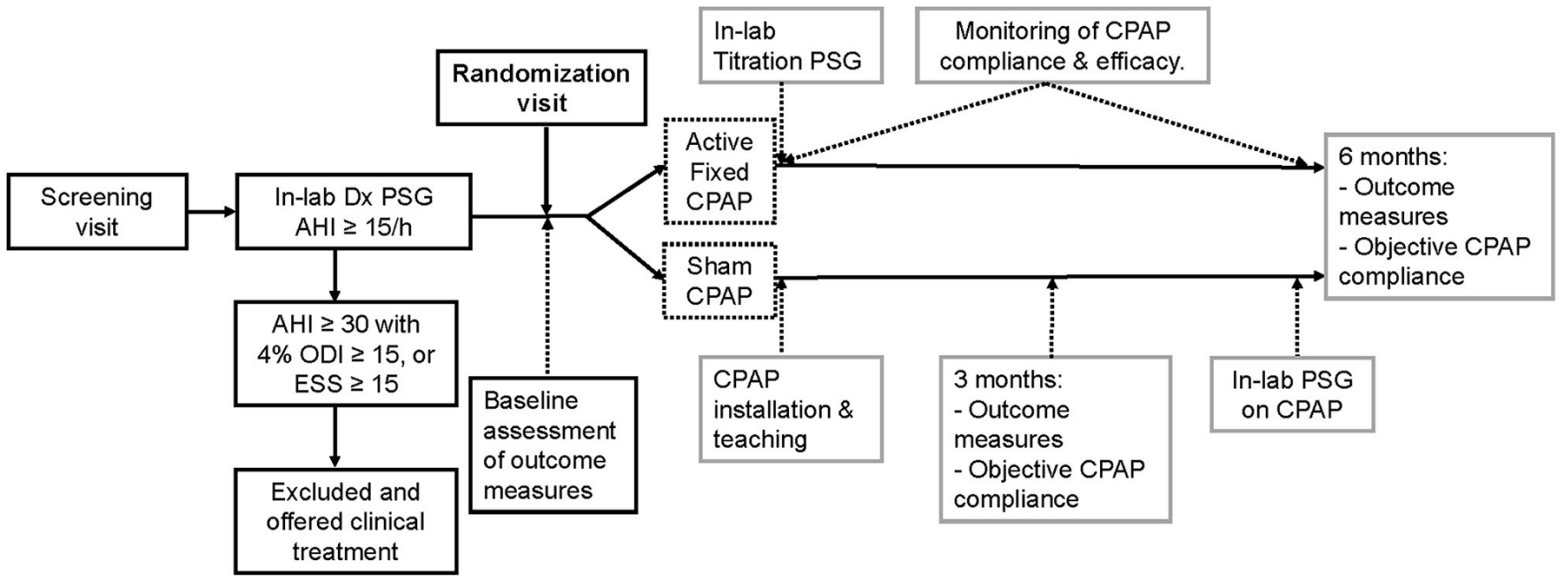
Study protocol. The figure outlines study procedures and visits from screening to study end at 6 months of treatment. Gray boxes indicate events and visit post-randomization relevant for both active and sham CPAP groups. Compliance with study CPAP machine was evaluated for both groups during the 6-month treatment phase of the study. ESS: Epworth Sleepiness Scale; PSG: polysomnography; AHI: apnea-hyponea index; ODI: oxygen desaturation index.

The study protocol is outlined in Figure 1. At screening, patients underwent a complete medical and standardized sleep history, including a question on morning fatigue (Are you fatigued on awakening in the morning?), Restless Legs Syndrome diagnostic questionnaire,^20^ physical exam by a study Neurologist or Physiatrist (P.D., Y.L., D.A.T.) with determination of EDSS, MoCA, PSQI, FSS, and ESS, and blood sampling. Eligible patients then underwent complete overnight in-laboratory PSG.^21^ The subsequent randomization visit included evaluation by Sleep specialists (J.K., S.K., and V.J.) and additional questionnaires including Fatigue Scale for Motor and Cognitive Functions (FSMC),^22^ pain visual analog scale (VAS) scores, Multiple Sclerosis Quality of Life-54 (MSQOL-54),^23^ Center for Epidemiological Studies-Depression Scale (CES-D),^24^ and Tower of London-Drexel University (TOLDX) assessment (evaluates executive function).^25^ After randomization, CPAP teaching, and installation, participants underwent manual CPAP titration PSG. Patients were instructed to use their CPAP nightly for 6 months. Study assessments were completed at 3 and 6 months and included an interval history, neurological exam with EDSS, relapse assessment, study questionnaires and assessments (MoCA, TOLDX, morning fatigue question only at 6 months), CPAP satisfaction and side effect questionnaire, CPAP compliance, and Sleep specialist interview. PSG on study CPAP machine was obtained prior to the final visit.

### CPAP treatment

Active and sham CPAP units (Philips Respironics System One) were identical in appearance. Active CPAP was set based on PSG titration with subsequent adjustment as needed to optimize download AHI. Objective compliance was determined regularly from the CPAP memory and recorded at 3 and 6 months. Patients with suboptimal compliance received intermittent phone calls; those with persistent difficulties were referred to a blinded clinical sleep nurse. A blinding questionnaire was completed at 6 months.

### Sleep studies

Complete overnight PSG was performed using standard methods as described previously.^8,9,26,27^ Data were acquired initially using Stellate systems (Natus, Inc., Harmonie 7.0 software), then from 2015 onward using Nihon-Kohden PSG 1100 (Polysmith v. software). PSG studies were scored manually by a certified technologist with sleep physician review using American Academy of Sleep Medicine (AASM) 2012 criteria,^28^ with the exception of respiratory events which were scored using Chicago criteria.^21^ Summary PSG outcome measures were generated as previously described.^8,9^ Manual CPAP titration studies were conducted using a standard approach^26^ utilizing the CPAP flow signal as the primary respiratory signal for effective CPAP and the mask pressure and respiratory inductance signals for sham CPAP.

### Sample size and statistical analysis

The primary outcome measure was the difference in FSS scores between baseline and 6 months of treatment. Based on our preliminary observational data for change in FSS following OSAH treatment in MS,^9^ considering a reported clinically important difference for FSS of 0.6 (95% CI, 0.3 to 0.9),^29^ we calculated that a sample size of 58 patients would enable us to detect a between-group difference in FSS of 0.9, with 80% power and alpha error of 0.05. Projecting a drop-out rate of 10%, we aimed to include a total of 65 patients.

**Figure 2.**
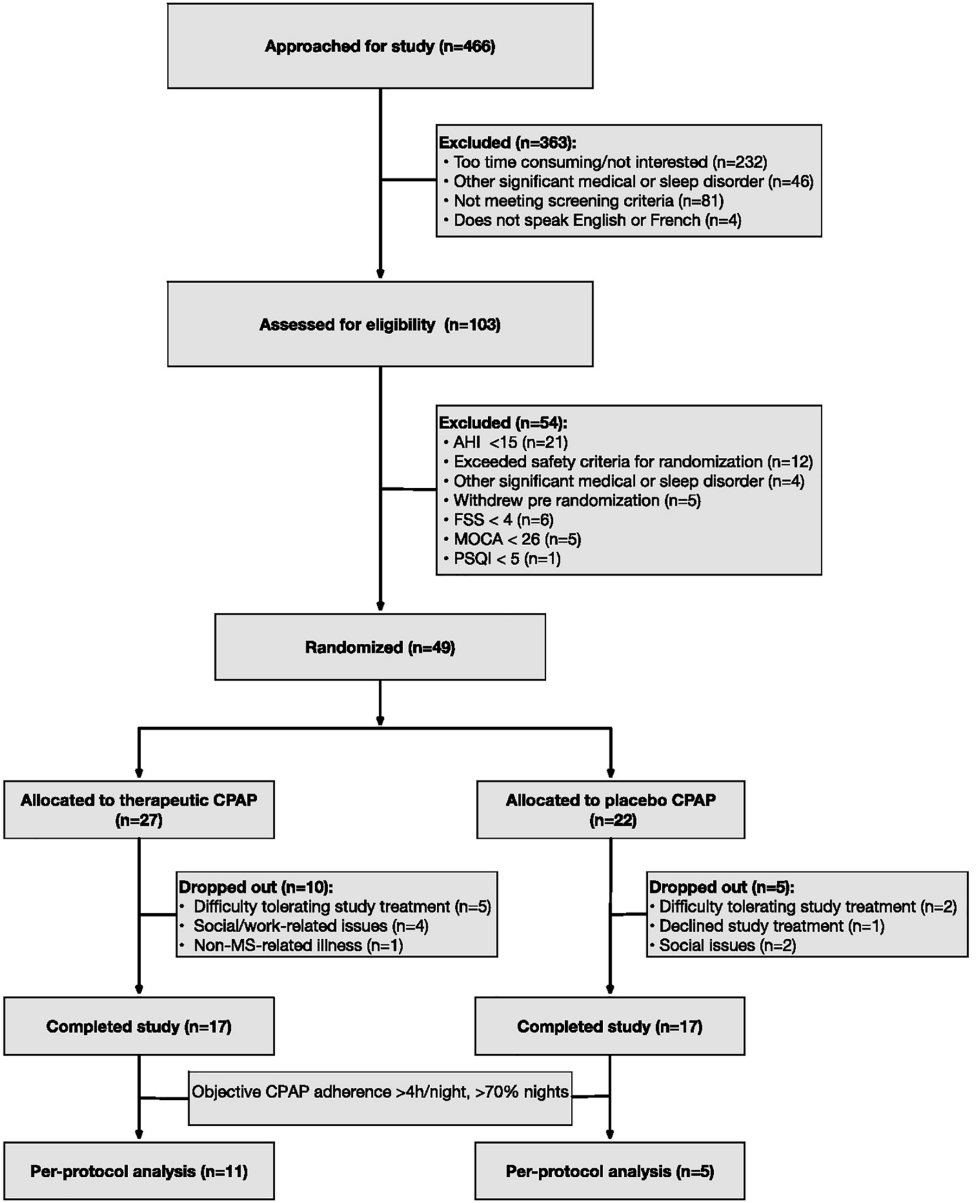
CONSORT flow diagram. Of the 466 patients approached for the study, 49 were randomized, 34 (17 in each group) completed the study protocol and were included in the primary study analysis. PSQI: Pittsburgh Sleep Quality Index; FSS: Fatigue Severity Scale; MoCA: Montreal Cognitive Assessment; AHI: apnea-hypopnea index.

The primary analysis compared mean changes in the FSS (primary outcome) between treatment and control groups between baseline and 6 months. Secondary analyses included a comparison of mean changes between treatment and control groups for the secondary outcomes (FSMC, ESS, PSQI, pain VAS scores, EDSS, MSQOL-54 (physical and mental health composite summary; PCS and MCS), CES-D, MoCA, TOLDX, PSG variables, and objective CPAP compliance) at 6 months, and a comparison of mean changes between groups of all outcomes (when available) at 3 months. Exploratory analyses included comparison of changes in remaining outcome measures (morning fatigue question from the standardized sleep questionnaire, CPAP satisfaction questionnaire, and MS relapses) between treatment groups. Our primary analysis included all patients who completed the study protocol. This approach was adapted post hoc due to early drop-out and extensive missing data from both arms for these drop-outs (Figure 2). (Intention to treat analysis was performed secondarily and is presented in Supplemental Materials.) Missing values for outcome measures were assigned using multiple imputation through chained equations.^30,31^ Continuous variables were evaluated with linear regression analysis and the binary outcome with the Wald test. The primary analysis was unadjusted, but a secondary analysis was adjusted for age and sex, but not other variables due to the relatively small number of patients. Another secondary analysis (“Per protocol analysis,” Figure 2) included only patients compliant with CPAP (using CPAP at least 4 hours per night for at least 70% of nights). Finally, in an exploratory analysis, mean disease alleviation (MDA) was calculated using the equation: [(diagnostic AHI – AHI on treatment)/Diagnostic AHI] × [CPAP Use (h/night)/Total Sleep Time].^32,33^ MDA was calculated by two methods: MDA_p_ using AHI on treatment obtained from end of study PSG and MDA_c_ calculated using AHI on treatment obtained from 6-month CPAP download. MDA_c_ was only calculated for the treatment group since for the sham CPAP group, there was no AHI obtainable from download. The association of MDA with primary and secondary outcomes was evaluated with an adjusted linear regression model.

All tests were two-sided, and a *p*-value less than 0.05 was considered to be statistically significant. We did not adjust for multiple analyses since we identified a single primary outcome a priori. The data were analyzed with RStudio statistical software.^34^

## Results

### Patients

Of the 466 patients approached for the study, 103 completed screening (Figure 2). The main reasons for declining participation were lack of time or interest (*n* = 232). In total, 49 patients fulfilled study criteria and were enrolled. Of the patients excluded, 21 had AHI < 15, 12 exceeded the AHI and other study safety criteria and were treated emergently. 27 patients were randomly assigned to fixed CPAP treatment and 22 to sham CPAP. However, 10 patients in the active treatment group and five controls either never used CPAP or used the machine one to two times and then dropped out of the study. In total, 17 patients in each group completed the study protocol and were included in the primary analysis. Recruitment was halted prior to achieving study target population due to the depletion of operating funds.

Clinical characteristics at baseline and during the study period of patients who completed the study were similar in both groups (Table 1). Most patients were on Fingolimod, Natalizumab, or other newer MS immunomodulating medications. Patients had few other comorbidities in addition to MS (Supplementary Table S1). There were no differences in baseline characteristics of patients who completed the study and all patients who were randomized (Supplemental Table S2). However, patients who dropped out had significantly lower EDSS, lower FSS, and less severe OSAH than study completers (Supplemental Table S3).

**Table 1.** Characteristics of study subjects according to treatment group.

Characteristic/outcome Mean ± SD, *N* (%)	Active CPAP (*n* = 17)	Sham CPAP (*n* = 17)
Characteristics at baseline		
Age (years)	49.6 ± 10	52.8 ± 8.8
Female sex	11 (64.7%)	11 (64.7%)
Body mass index (kg/m^2^)	28.8 ± 6.7	30.0 ± 7.6
MS disease duration (years)	21.2 ± 10.1	19.8 ± 11
MS type		
Relapsing remitting	14 (82.4%)	13 (76.5%)
Secondary progressive	3 (18.8%)	4 (23.5%)
Immunomodulating treatment at baseline	11 (64.7%)	10 (58.8%)
EDSS	4.2 ± 1.8	3.9 ± 1.5
Characteristics during the study period		
Change in MS immunomodulating treatment	2 (11.8%)	1 (5.9%)
MS exacerbations	3 (17.6%)	3 (17.6%)
Initiated steroids	1 (5.9%)	2 (11.8%)

CPAP: continuous positive airway pressure; SD: standard deviation; EDSS: Expanded Disability Status Scale; MS: multiple sclerosis.

**Table 2.** Changes in outcome measures according to treatment group at 3 and 6 months (study completers).

Variable	Active CPAP (*n* = 17)			Sham CPAP (*n* = 17)			*p*-values	
	Baseline	3 months	6 months	Baseline	3 months	6 months	3 months	6 months
Fatigue severity scale	6.2 ± 0.7	5.3 ± 1.1	5.7 ± 0.9	5.7 ± 0.8	5.4 ± 0.81	5.4 ± 0.9	**0.09**	0.64
FSMC								
Total score	74.5 ± 13.3	70.4 ± 13.9	71.7 ± 16.1	71.9 ± 17.8	69.4 ± 15.8	67.7 ± 13.5	0.65	0.80
Cognitive score	35.4 ± 8.3	34.4 ± 7.5	34.5 ± 9.6	34.7 ± 9.0	34.2 ± 7.9	33.1 ± 6.5	0.89	0.81
Motor score	39.5 ± 6.8	36.7 ± 7.3	37.2 ± 7.8	36.8 ± 9.0	35.1 ± 9.1	34.6 ± 7.9	0.64	0.95
Morning fatigue (proportion)	0.88		0.47	0.82		0.82		**0.03**
PSQI	9.9 ± 3.5	8.5 ± 3.9	8.1 ± 4.3	11.9 ± 3.4	11.0 ± 3.0	10.0 ± 3.2	0.59	0.92
ESS	12.5 ± 4.8	9.12 ± 4.9	10.5 ± 4.2	8.7 ± 4.6	7.94 ± 4.8	6.6 ± 4.0	**0.03**	0.93
EDSS	4.2 ± 1.8	4.3 ± 1.4	4.2 ± 1.5	3.9 ± 1.5	4.2 ± 1.4	3.9 ± 1.5	0.78	0.78
Pain due to illness (VAS)	37.5 ± 27.8	44.7 ± 30	37.7 ± 31.6	40.8 ± 25.7	33.4 ± 25.2	34.9 ± 30.0	**0.09**	0.52
Night pain (VAS)	34.1 ± 32.4	36.4 ± 35	29.2 ± 29.0	33.7 ± 22.9	33.5 ± 30.40	29.9 ± 29.1	0.81	0.93
CES-D	20.3 ± 5.2	19.2 ± 6.1	19.4 ± 6.5	22.7 ± 5.7	19.6 ± 4.5	19.9 ± 5.0	0.31	0.19
MSQOL-54								
Physical composite score	42.8 ± 11.5	41.3 ± 11.4	43.2 ± 11.3	42.0 ± 14.3	43.1 ± 14.3	44.2 ± 13.7	0.38	0.53
Mental composite score	35.4 ± 9.0	35.5 ± 3.4	34.3 ± 9.0	33.4 ± 7.5	36.5 ± 7.7	38.8 ± 5.3	0.10	**<0.01**
MoCA	2 8 ± 1.4		27.9 ± 2.0	28.1 ± 1.5		27.8 ± 2.0		0.80
Tower of London								
Total correct score	5.06 ± 2.5		6.1 ± 2.1	5.4 ± 2.0		6.0 ± 2.1		0.56
Standard total correct score	105.8 ± 14.5		113.0 ± 13.3*	111.5 ± 15.9		115.4 ± 16.7		0.45
Total move score	25.4 ± 16.6		19.0 ± 15	26.2 ± 22.2		2.8 ± 14.9		0.62
Standard move score	105.7 ± 14.7		109.6 ± 12.7	107.0 ± 16.4		108.9 ± 14.0		0.65
Total time (minutes)	328.8 ± 153.4		267.1 ± 101 7	324.0 ± 149.71		279.8 ± 96.4		0.69
Standard total time	94.9 ± 17.26		100.8 ± 12.4	96.12 ± 14.6		100.2 ± 12.0		0.68

CPAP: continuous positive airway pressure; FSMC: Fatigue Scale for Motor and Cognitive Functions; PSQI: Pittsburgh Sleep Quality Index; ESS: Epworth Sleepiness Scale; EDSS: Expanded Disability Status Scale; VAS: visual analog scale; CES-D: Centers for Epidemiological Studies–Depression Scale; MSQOL-54: Multiple Sclerosis Quality of Life-54; MoCA: Montreal Cognitive Assessment.

Values are mean ± standard deviation unless otherwise indicated. *p*-values presented are for the difference in change between baseline and 3- or 6-month assessment for the two treatment groups.

* The change within this group showed a trend (*p* = 0.09) to improvement.The *p*-values marked in bold are statistically significant.

### Outcome analyses

For the primary outcome of change in FSS at 6 months, the study showed no difference between active and sham CPAP groups (*p* = 0.64), with a mean reduction of FSS between baseline and 6 months of 0.32 in the treatment group and 0.2 in the control group (Table 2). There were no significant differences between treatment and control groups at 6 months with respect to the secondary outcomes of fatigue as assessed by the FSMC, somnolence (ESS), sleep quality (PSQI), pain (pain VAS), EDSS, PCS score of the MSQOL-54, depressed mood (CES-D), MoCA, and TOLDX (Table 2). For the secondary outcomes at 6 months, there was a slight improvement in mental quality of life in the control group which had a mean improvement of 5.82 in the MCS score of the MSQL-54 compared to −0.75 in the treatment group (*p* < 0.01, 95% CI of difference 2.85, 9.83). At 3 months, for secondary outcomes, we found a statistically and clinically^35^ significant reduction in mean sleepiness measured by ESS of 3.4 in the treatment group compared to 0.8 in the control group (*p* = 0.03, 95% CI of difference 0.34, 5.20). There was a clinically significant^29^ trend for improvement in the FSS with treatment with a reduction of mean 0.9 compared to 0.3 in controls (*p* = 0.09, 95% CI of difference −0.23, 1.20). At 3 months, there were no other significant differences between groups for outcome measures (Table 2). The adjusted analyses were similar to unadjusted analyses.

When all patients including drop-outs were included in an intention-to-treat (ITT) analysis, the results for the primary and secondary outcomes were similar to the results in study completers (Supplemental Table S4).

PSG data are presented in Table 3. Baseline PSG characteristics between groups were similar in both groups (Table 3). With treatment, there was a significant reduction in the mean total arousal index, AHI, obstructive hypopnea index, and 4% oxygen desaturation index, and a trend to increased total sleep time in the active CPAP group only. Sham CPAP did not yield significant changes in PSG parameters. AHI reduction was significantly greater with active CPAP compared to sham.

**Table 3.** Baseline diagnostic and 6-month on-CPAP polysomnographic data.

Variable	Active CPAP (*n* = 17)			Sham CPAP (*n* = 17)			*p*-value
	Baseline diagnostic	6 months on CPAP	*p*-value	Baseline diagnostic	6 months on CPAP	*p*-value	
Total sleep time (hours)	5.5 ± 1.0	6.2 ± 1.0	0.06	5.2 ± 0.9	5.4 ± 1.4	0.70	**<0.05**
Sleep efficiency (%)	75.9 ± 13.4	81.6 ± 12.7	0.23	75.6 ± 10.7	77.3 ± 19.1	0.75	0.43
Wake after sleep onset (minutes)	88.4 ± 52.8	70.8 ± 59.9	0.36	76.2 ± 44.8	77.4 ± 60.7	0.92	0.32
Arousal index (*n*/hour)	47.5 ± 17.4	33.0 ± 13.1	**0.01**	55.4 ± 26.9	44.8 ± 15.5	0.17	0.62
Stage N1 (% TST)	18.2 ± 9.8	13.7 ± 7.8	0.17	22.4 ± 14.8	19.3 ± 10.0	0.53	0.70
Stage N2 (% TST)	52.5 ± 12.4	51.8 ± 12.3	0.80	50.2 ± 10.7	46.1 ± 17.2	0.79	1.00
Stage N3 (% TST)	16.2 ± 11.6	18.1 ± 11.7	0.65	16.2 ± 16.6	15.8 ± 13.4	0.94	0.74
Stage R (% TST)	13.1 ± 7.3	16.3 ± 8.8	0.26	11.3 ± 5.3	14.3 ± 7.0	0.11	0.91
Apnea-hypopnea index (*n*/hour)	31.0 ± 11.5	12.4 ± 6.9	**<0.01**	36.0 ± 24.9	25.2 ± 14.1	0.13	0.28
Obstructive hypopnea index (*n*/hour)	29.2 ± 11.6	10.9 ± 6.3	**0.01**	34.3 ± 20.5	23.8 ± 13.7	0.41	**<0.01**
4% oxygen desaturation index (*n*/hour)	4.7 ± 4.6	1.1 ± 1.3	**<0.01**	2.8 ± 3.8	4.2 ± 6.0	0.14	0.26
Nadir SaO_2_ (%)	85.6 ± 9.5	89.7 ± 3.9	0.13	86.9 ± 4.1	86.7 ± 5.1	0.91	0.06

CPAP: continuous positive airway pressure; TST: total sleep time; SaO_2_: hemoglobin oxygen saturation.

Values are mean ± standard deviation.

*p*-values presented are for the differences in change between baseline and 6 months for the two treatment groups.

Statistically significant p-values are marked in bold.

### CPAP compliance

The active treatment group had better overall compliance than the control group (Table 4). Percent days with the usage for more than 4 hours were a mean of 70% in the active CPAP group and 35% in the sham group. In total, 11 patients in the treatment group and five controls achieved the minimum requirement of per protocol CPAP usage of at least 4 hours/night for 70% of nights.

**Table 4.** CPAP adherence and efficacy according to treatment group.

Variable	Active CPAP (*n* = 17)	Sham CPAP (*n* = 17)
Usage all nights (hours/night)	5.5 ± 2.8	2.8 ± 2.1
Usage on nights used (hours/night)	6.3 ± 2.2	4.1 ± 2.1
Percent nights with > 4 hours use	69.6 ± 32.7	34.7 ± 31.8
Mean pressure (cm H_2_O)	9.0 ± 2.6	N/A
Residual AHI (*n*/hour)	1.7 ± 1.4	N/A
Mean disease alleviation		
Based on final PSG AHI, TST (%)	52.4 ± 35.5	0.7 ± 42.5
Based on CPAP microprocessor AHI, PSQI TST (%)	82.9 ± 35.0	N/A

CPAP: continuous positive airway pressure; AHI: apnea-hypopnea index; PSG: polysomnogram; TST: total sleep time; PSQI: Pittsburgh Sleep Quality Index.

### Exploratory analyses

In an exploratory outcome, on a standardized sleep questionnaire, a significant proportion of patients in the treatment group reported resolution of morning fatigue compared to the control group at 6 months (*p* = 0.03) (Table 2). There was no difference between treatment and control groups for the exploratory outcome of MS relapses.

On the exploratory outcome of the CPAP satisfaction questionnaire, at 6 months, patients in the fixed CPAP group were significantly more likely to report that they were better off since they started using CPAP than controls (*p* = 0.04). In addition, patients in the fixed CPAP group tended to report more frequently that they wished to use CPAP in the long run than controls (*p* = 0.06).

### Blinding

The chi-square *p*-value comparing the proportions of patients and investigators guessing that patients were on active treatment provided evidence for unblinding for patients but not investigators (*p* = 0.0028 and *p* = 0.16) (Supplemental Table S5).

### Adverse effects

Adverse effects were related to the physical effects of the machine and mask. Patients in the sham CPAP group had more difficulty waking up during the night due to CPAP and those using active CPAP had more difficulty with air leaking around the mask, but otherwise side effects were similar in the two groups (Table 5).

**Table 5.** Adverse effects at 6 months.

Adverse effect	Active CPAP	Sham CPAP	*p*-value
Difficulty donning/doffing gear	3 (17.6)	6 (35.3)	0.44
Difficulty finding a comfortable sleeping position due to the mask/tubing	11 (64.7)	13 (76.5)	0.71
Nose congested or runny	7 (41.2)	9 (52.9)	0.73
Nosebleed, or nose dry or irritated	3 (17.6)	5 (29.4)	0.93
Mouth dry	8 (47.1)	11 (64.7)	0.49
Difficulty with air pressure	2 (11.8)	7 (41.2)	0.12
Difficulty with machine noise	4 (23.5)	5 (29.4)	1.00
Difficulty with air leaking from around the mask	12 (70.6)	5 (29.4)	**0.04**
Difficulty with air escaping through your mouth	7 (41.2)	3 (17.6)	0.26
Difficulty waking up during the night due to CPAP	5 (29.4)	12 (70.6)	**0.04**
CPAP bothers my bed partner	6 (35.3)	4 (23.5)	0.71

CPAP: continuous positive airway pressure.

Values presented are n’s and percentages in parentheses. Statistically significant p-values are marked in bold.

### Analysis in compliant patients

For the subset of patients meeting threshold CPAP compliance (Figure 2), there were no significant beneficial effects of active CPAP compared with sham on primary and secondary outcome measures (Supplemental Table S6).

### MDA

MDA_p_ (mean ± SD) was 52.4% ± 35.5% in the active group and 0.73% ± 42.5% in the control group (Table 4). Results of MDA were similar when TST from the PSQI was used to calculate MDA. However, MDA_c_ for patients in the treatment group was higher at 85.5% ± 38.8%. There was no association of MDA_P_ and FSS at 3 and 6 months in CPAP-treated patients, and no association between the change in FSS and MDA_P_ at 3 and 6 months (Supplemental Table S7). However, at 3 and 6 months, reduced PSQI score was significantly associated with improved MDA_p_ (*p* = 0.01 and *p* < 0.01, respectively).

## Discussion

The results of this two-center, randomized, sham-CPAP-controlled clinical trial of a 6-month course of CPAP in MS patients with OSAH demonstrate no statistically significant beneficial effect of the treatment on the predefined primary outcome of the FSS, and secondary outcomes of FSMC, sleep quality, somnolence, pain, disability, and quality of life at 6 months. However, at 3 months of treatment, for secondary outcomes, we observed a trend to a reduction of fatigue and a significant reduction in somnolence with CPAP. Both improvements would be deemed clinically important, but may have been influenced by unblinding of subjects. In an exploratory analysis, there was a significant reduction in the proportion of patients reporting morning fatigue with active CPAP treatment. In another exploratory analysis, patients were more likely to believe they were better off with active compared with sham CPAP treatment. Despite some positive results from secondary and exploratory analyses of clinical data and objective improvement of sleep on PSG, this study was formally negative and did not show a clear benefit of CPAP on the primary and secondary clinical outcomes at 6 months. Therefore, the possible effect of CPAP on clinical symptoms in this population of patients requires further study.

The finding of no statistically significant beneficial effects of CPAP on predefined clinical outcomes in MS patients with OSAH at 6 months does not reflect the previous experience in observational studies^9,13^ which showed a clinical benefit with CPAP in a proportion of patients. Several potential study limitations could contribute to this discrepancy. First, we excluded patients with severe OSAH for ethical and safety reasons. However, these patients may have potentially benefited most from the treatment. Second, we had a limited sample size and did not obtain our target patient population, which was at least in part related to the complexity of the protocol with significant patient burden and 6-month duration. Third, despite a concerted effort of the study team, treatment compliance was not optimal, reducing the chance of finding a treatment effect. Of the 49 randomized patients, 15 dropped out soon after randomization with difficulty tolerating CPAP. Since these patients were less disabled and had less severe OSAH, our results could be influenced by their exclusion from the analysis. Of the 34 patients who completed the study, relatively few (Figure 2) met threshold CPAP compliance criteria in both groups. Thus, the impact of OSAH treatment may have been limited by suboptimal compliance. Fourth, the inclusion of patients with elevated PSQI scores is another potential limitation which could have affected our findings, although only one potential study subject who met other eligibility criteria was excluded on this basis. Finally, our primary outcome of fatigue is subjective and is influenced by many factors in MS.^3^ A change in other factors can impact a patient’s fatigue level and may make it difficult to identify a change due to OSAH treatment. In addition, our primary measure, the FSS, did not include a time of day component which would be important for future studies due to our finding of a significant reduction in morning fatigue with CPAP.

Our study has several strengths. It is the first randomized, placebo-controlled, double-blinded study of OSAH treatment for MS fatigue. The control group used sham CPAP which helped reduce bias. We targeted patients with fatigue and poor sleep quality which would be expected to aid in identifying a treatment effect. In addition, we had a relatively long intervention period of 6 months which we believed would provide sufficient time for acclimatization to therapy, and to observe any changes in clinical symptoms. However, based on our experience, an even longer period may be necessary for MS patients due to the time necessary to become acclimatized to CPAP and to address any difficulties, such as with mask comfort.

In conclusion, this randomized, double-blinded, sham CPAP-controlled clinical trial of CPAP treatment of MS patients with fatigue and non-severe OSAH did not show a significant improvement in the primary outcome measure of FSS at 6 months. We did observe some improvements in secondary outcome measures of fatigue and somnolence at 3 months, and a significant resolution of morning fatigue, an exploratory outcome, at 6 months with CPAP. The latter results should be viewed with caution. This study does not exclude a potential beneficial effect of CPAP in MS. We believe that further studies are warranted in this area given the potential impact for patients. Future studies should include longer term evaluation of outcomes, alternate fatigue assessment instruments, larger sample sizes, use of sleep hygiene measures with OSAH treatment, and the inclusion of patients with more severe OSAH. In addition, other therapeutic approaches for OSAH in MS should be considered.

## Supplemental Material

sj-pdf-1-msj-10.1177_13524585211010390 – Supplemental material for Effect of continuous positive airway pressure treatment of obstructive sleep apnea-hypopnea in multiple sclerosis: A randomized, double-blind, placebo-controlled trial (SAMS-PAP study) Click here for additional data file. Supplemental material, sj-pdf-1-msj-10.1177_13524585211010390 for Effect of continuous positive airway pressure treatment of obstructive sleep apnea-hypopnea in multiple sclerosis: A randomized, double-blind, placebo-controlled trial (SAMS-PAP study) by Sulaiman Khadadah, R John Kimoff, Pierre Duquette, Vincent Jobin, Yves Lapierre, Andrea Benedetti, Fatema T Johara, Ann Robinson, Elaine Roger, Amit Bar-Or, Gabriel Leonard, Marta Kaminska and Daria A Trojan in Multiple Sclerosis Journal
