# Supplementary material for: Effect of continuous positive airway pressure treatment of obstructive sleep apnea-hypopnea in multiple sclerosis: A randomized, double-blind, placebo-controlled trial (SAMS-PAP study)
Source: Mult Scler. 2021 Apr 23;28(1):82–92. doi: 10.1177/13524585211010390 (PMC8688981; doi:10.1177/13524585211010390)
Supplement: sj-pdf-1-msj-10.1177_13524585211010390 – Supplemental material for Effect of continuous positive airway pressure treatment of obstructive sleep apnea-hypopnea in multiple sclerosis: A randomized, double-blind, placebo-controlled trial (SAMS-PAP study) [file sj-pdf-1-msj-10.1177_13524585211010390.pdf]

Supplementary Data for SAMS-PAP Study

TABLE S1. MEDICAL CONDITIONS IN PATIENTS WHO COMPLETED THE STUDY 2

TABLE S2. BASELINE CHARACTERISTICS OF STUDY COMPLETERS COMPARED TO ALL RANDOMIZED PATIENTS 3

TABLE S3. BASELINE CHARACTERISTICS OF STUDY COMPLETERS COMPARED TO PATIENTS WHO DROPPED OUT……………………………………………………………….5

TABLE S4. CHANGES IN OUTCOME MEASURES ACCORDING TO TREATMENT GROUP AT THREE AND SIX MONTHS IN ALL RANDOMIZED PATIENTS (INTENTION TO TREAT ANALYSIS) 7

TABLE S5. BLINDING OUTCOMES……………………………………………………………….9

TABLE S6. CHANGES IN OUTCOME MEASURES ACCORDING TO TREATMENT GROUP AT SIX MONTHS IN COMPLIANT PATIENTS…………………………………………………………10

TABLE S7. LINEAR REGRESSION MODELS OF EFFECT OF MDAP ON GSS AND CHANGE IN FSS, USING TOTAL SLEEP TIME (TST) FROM PSG AND USING SLEEP TIME FROM PSQI…11

Table S1. Medical Conditions in Patients Who Completed the Study

**Medical Condition - % (n)**	**Completed protocol (n=34)**	**Treatment group (n=17)**	**Control group (n=17)**
Hypertension	14.7 (5)	23.5 (4)	5.9 (1)
Cardiac disease	2.9 (1)	0 (0)	5.9 (1)
Diabetes mellitus	5.9 (2)	0 (0)	11.8 (2)
Migraine	20.6 (7)	29.4 (5)	11.8 (2)
Respiratory disease	14.7 (5)	23.5 (4)	5.9 (1)
Dyslipidemia	11.8 (4)	17.6 (3)	5.9 (1)
Hypothyroidism	11.8 (4)	17.6 (3)	5.9 (1)
Depression / Anxiety	29.4 (10)	29.4 (5)	29.4 (5)

Table S2. Baseline Characteristics of Study Completers Compared to All Randomized Patients

Baseline characteristic (Mean ± SD)	All Randomized patients (n=49)	Study Completers (n=34)	Randomized treatment group (n=27)	Randomized control group (n=22)
Age	48 ± 13.6	47.4 ± 15	49.9 ± 10.6	45.7 ± 16.7
BMI	29 ± 6.7	26.9 ± 11.1	28.9 ± 6.3	29.2 ± 7.3
Female sex - %(n)	67.3 (33)	64.7 (22)	66.7 (18)	68.2 (15)
**Subtype of MS - %(n):**				
Relapsing/Remitting	81.6 (40)	79.4 (27)	85.2 (25)	77.3 (17)
Secondary Progressive	14.3 (7)	20.6 (7)	11.1 (3)	18.2 (4)
Primary Progressive	4.1 (2)	0 (0)	3.7 (1)	4.5 (1)
Progressive Relapsing	0 (0)	0 (0)	0 (0)	0 (0)
MS disease duration	5.1 ± 4.8	5.4 ± 5.2	4.8 ± 4.1	5.4 ± 5.9
Current immunomodulating MS treatment- %(n)	49 (24)	61.8 (21)	51.9 (14)	45.5 (10)
EDSS	3.6 ± 1.6	4 ± 1.7	3.5 ± 1.8	3.8 ± 1.4
ESS	10.4 ± 4.8	10.6 ± 5	11.6 ± 4.4	8.8 ± 4.9
FSS	5.8 ± 0.9	5.9 ± 0.8	5.8 ± 1.0	5.8 ± 0.7
MoCA	28.1 ± 1.4	28.1 ± 1.5	28.1 ± 1.4	28.1 ± 1.5
VAS pain	39.6 ± 27.4	39.1 ± 26.4	35.5 ± 27.0	44.5 ± 27.7
VAS night pain	34.6 ± 29	33.9 ± 27.6	33.2 ± 31.6	36.3 ± 26.0
PSQI	10.9 ± 3.6	10.9 ± 3.5	10 ± 3.4	12 ± 3.6
**MSQOL-54:**				
Physical health composite	44.1 ± 12.4	42.3 ± 12.8	35.7 ± 8.5	33.8 ± 7.4
Mental health composite	34.9 ± 8	34.4 ± 8.2	10.8 ± 4.5	9.2 ± 3
**CES-D**	21.5 ± 5.2	21.5 ± 5.5	20.9 ± 4.8	22.1 ± 5.7
**FMCS:**				
Combined motor and cognitive score	71.9 ± 14.7	72.6 ± 15.7	71.4 ± 13.0	72.5 ± 16.9
Cognitive score	34.6 ± 8.2	34.7 ± 8.7	33.8 ± 7.8	35.5 ± 8.8
Motor score	37.3 ± 7.5	37.8 ± 8.1	37.6 ± 6.6	37.0 ± 8.6
**Baseline PSG data:**				
Total Sleep Time (TST)(min)	323.2 ± 58	321.3 ± 56.9	325.2 ± 56.1	320.7 ± 61.5
Sleep Efficiency (SE) (%)	76.2 ± 12.3	75.7 ± 11.9	76 ± 13	76.4 ± 11.7

Respiratory Arousal index (/h)	38.1 ± 36.3	32 ± 21.1	31 ± 19.5	46.9 ± 48.9
Total Arousal index (/h)	51.1 ± 21.6	51.5 ± 22.7	48.4 ± 19.5	54.4 ± 23.9
Periodic Limb movement (PLM) index (/h)	9.8 ± 17.2	8.5 ± 14.1	7.8 ± 14.2	12.5 ± 20.6
Mean SaO2 during sleep (%)	94.4 ± 2.4	94.3 ± 2.7	94.4 ± 3.1	94.5 ± 0.9
Min SaO2 % during sleep (%)	88.4 ± 8.2	87.4 ± 9.3	88.4 ± 10.6	88.4 ± 4.6
Apnea Hypopnea index (AHI) (/h)	32.1 ± 17.5	33.5 ± 19.3	30.8 ± 12.4	33.8 ± 22.5
4% oxygen desaturation index (ODI) (/h)	3.6 ± 4	3.8 ± 4.3	4.6 ± 4.2	2.5 ± 3.4
Central Apnea index (/h)	0.7 ± 2	0.4 ± 0.4	0.8 ± 2.6	0.5 ± 0.5
Mixed Apnea index (/h)	0.3 ± 0.8	0.2 ± 0.3	0.4 ± 1	0.1 ± 0.2
Obstructive Apnea index(/h)	0.9 ± 1.6	1.1 ± 1.7	1 ± 1.6	0.8 ± 1.6
Central Hypopnea index (/h)	0.1 ± 0.2	0.1 ± 0.2	0.1 ± 0.2	0.1 ± 0.2
Obstructive Hypopnea index (/h)	30.2 ± 16.9	31.8 ± 19.1	28.4 ± 11.2	32.3 ± 22.2

Legend: Values presented are mean ± SD. Fatigue severity scale (FSS), Fatigue Scale for Motor and Cognitive Functions (FMCS), Epworth sleepiness scale ESS, Pittsburgh Sleep Quality Index (PSQI), Pain visual analog scale (VAS), MS-specific quality of life measure-54 (MSQOL-54), Expanded Disability Status Scale score (EDSS), Center for Epidemiological Studies-Depression Scale (CES-D), Montreal Cognitive Assessment (MoCA).

Table S3. Baseline Characteristics of Study Completers Compared to Patients Who Dropped Out

**Baseline characteristic (Mean ± SD)**	**Study Completers (n=34)**	**Patients Who Dropped Out (n=15)**	**P-Values**
Age	47.4 ± 15	49.3 ± 10.5	0.55
BMI	26.9 ± 11.1	28.0 ± 4.2	0.38
Female sex - %(n)	64.7 (22)	73.2 (11)	1
**Subtype of MS - %(n):**			
Relapsing/Remitting	79.4 (27)	86.7 (13)	0.71
Secondary Progressive	20.6 (7)	0	0.68
Primary Progressive	0 (0)	13.3 (2)	1
Progressive Relapsing	0 (0)	0 (0)	
MS disease duration	5.4 ± 5.2	4.0 ± 4.2	0.22
Current immunomodulating MS treatment- %(n)	61.8 (21)	53.3 (8)	0.76
EDSS	4 ± 1.7	2.7 ± 1.1	0.002
ESS	10.6 ± 5	9.9 ± 4.4	0.62
FSS	5.9 ± 0.8	5.3 ± 0.9	0.03
MoCA	28.1 ± 1.5	28.1 ± 1.4	0.87
VAS pain	39.1 ± 26.4	40.5 ± 30.6	0.88
VAS night pain	33.9 ± 27.6	36.3± 32.8	0.81
PSQI	10.9 ± 3.5	10.9 ± 4.0	0.95
**MSQOL-54:**			
Physical health composite	42.3 ± 12.8	48.3 ± 10.5	0.1
Mental health composite	34.4 ± 8.2	36.0 ± 7.8	0.52
**CES-D**	21.5 ± 5.5	21.5 ± 4.5	1
**FMCS:**			
Combined motor and cognitive score	72.6 ± 15.7	70.5 ±12.7	0.63
Cognitive score	34.7 ± 8.7	34.2 ±7.5	0.83
Motor score	37.8 ± 8.1	36.3 ± 5.9	0.45
**Baseline PSG data:**			
Total Sleep Time (TST)(min)	321.3 ± 56.9	319.3 ± 64.8	0.94
Sleep Efficiency (SE) (%)	75.7 ± 11.9	75 ± 14.3	0.84

Respiratory Arousal index (/h)	32 ± 21.1	24.0 ± 9.3	0.02
Total Arousal index (/h)	51.5 ± 22.7	49.3 ± 21.5	0.76
Periodic Limb Movements (PLM) during sleep index (/h)	8.5 ± 14.1	14.8 ± 25.3	0.45
Mean SaO2 during sleep (%)	94.3 ± 2.7	95.1 ± 1.6	0.39
Min SaO2 % during sleep (%)	87.4 ± 9.3	91.3 ± 2.2	0.09
Apnea Hypopnea index (AHI) (/h)	33.5 ± 19.3	23.9 ± 7.3	0.02
4% oxygen desaturation index (ODI) (/h)	3.8 ± 4.3	3.4 ± 3.1	0.49
Central Apnea index (/h)	0.4 ± 0.4	0.5 ± 0.5	0.47
Mixed Apnea index (/h)	0.2 ± 0.3	0.1 ± 0.1	0.07
Obstructive Apnea index(/h)	1.1 ± 1.7	0.1 ± 0.2	0.003
Central Hypopnea index (/h)	0.1 ± 0.2	0.1 ± 0.1	0.66
Obstructive Hypopnea index (/h)	31.8 ± 19.1	23.2 ± 7.5	0.04

Legend: Values presented are mean ± SD. Fatigue severity scale (FSS), Fatigue Scale for Motor and Cognitive Functions (FMCS), Epworth sleepiness scale ESS, Pittsburgh Sleep Quality Index (PSQI), Pain visual analog scale (VAS), MS-specific quality of life measure-54 (MSQOL-54), Expanded Disability Status Scale score (EDSS), Center for Epidemiological Studies-Depression Scale (CES-D), Montreal Cognitive Assessment (MoCA).

Table S4. Changes in Outcome Measures According to Treatment Group at Three and Six Months in All Randomized Patients (Intention to Treat Analysis)

	Randomized to Fixed CPAP (n=27)			Randomized to Sham CPAP (n=22)			P-Values*	
	**Baseline**	**3 months**	**6 months**	**Baseline**	**3 months**	**6 months**	**3 Mos**	**6 Mos**
FSS	5.76 ± 1.0	5.27 ± 1.0	5.59 ± 0.9	5.75 ± 0.71	5.52 ± 0.8	5.33 ± 0.8	0.35	0.59
FSMC								
Total score	72.04 ± 32.2	67.4 ± 13.9	67.89 ± 16.3	72.9 ± 16.6	70.0 ± 15.3	69.73 ± 15.8	0.62	0.95
Cognitive score	33.96 ± 7.7	33.4 ± 7.3	32.56 ± 9.0	34.32 ± 10.2	34.9 ± 7.3	34.5 ± 7.6	0.95	0.95
Motor score	37.67 ± 6.5	35.6 ± 6.6	34.07 ± 8.1	37.45 ± 8.6	35.7 ± 9.4	36.64 ± 8.9	0.83	0.58
Morning fatigue (proportion)	0.91		0.55	0.84		0.84		**0.03**
PSQI	10 ± 3.4	8.93 ± 4.7	9.56 ± 5.0	12.05 ± 3.6	12.1 ± 3.5	9.86 ± 3.5	0.22	0.19
ESS	11.63 ± 4.4	8 ± 4.7	9.48 ± 4.8	8.68 ± 4.8	8 ± 4.3	7.41 ± 4.3	**0.01**	0.57
EDSS	3.52 ± 1.8	3.85 ± 1.3	3.72 ± 1.5	3.77 ± 1.4	3.89 ± 1.4	3.89 ± 1.3	0.26	0.36
Pain due to illness (VAS)	35.52 ± 27.0	46.3 ± 28.1	35.56± 33.7	44.5 ± 27.7	34.7 ± 26.2	35.59 ± 32.2	**0.01**	0.14
Night pain (VAS)	33.22 ± 31.6	39.2 ± 33.3	32.15 ± 30.9	36.32 ± 26.0	25.0 ± 25.4	30.36 ± 28.3	**0.03**	0.39
CES-D	20.93 ± 4.8	19.1 ± 5.9	20.3 ± 6.1	22.14 ± 5.7	20.5 ± 4.5	20.09 ± 5.0	0.93	0.55
MSQOL-54								
Physical Composite Score	44.27 ± 11.0	36.9 ± 13.1	46.6 ± 11.9	43.99 ± 14.1	39.7 ± 12.2	44.3 ± 12.7	0.46	0.85
Mental Composite Score	35.75 ± 8.6	37.1 ± 8.6	36.16 ± 9.3	33.79 ± 7.4	37.0 ± 7.7	37.93 ± 5.4	0.25	**0.02**
MoCA	28.04 ± 1.4		27.81 ± 1.9	28.09 ± 1.5		27.68 ± 1.89		1.00
**Tower of London:**								
total correct score	6.67 ± 11.1		5.52 ± 2.4	5.5 ± 1.9		6.18 ± 2.0		0.40
Standard correct score	106 ± 23.4		110.59 ± 16.5	111.82 ± 14.2		116.82 ± 15.9		0.88
Total move score	31.19 ± 19.1		24.04 ± 17.4	26.09 ± 19.7		22.32 ± 14.5		0.51
Standard move score	100.74 ± 16.6		101.33 ± 32.0	129.64 ± 96.7		111.09 ± 13.6		0.26
Total time (mins)	324.1 ± 139.9		258.3 ± 94.6	320 ± 138.4		281.82 ± 90.2		0.48
Standard time	95.11 ± 15.2		102.07 ± 11.3	96.27 ± 14.4		98.82 ± 13.2		0.29

Legend: Missing values for outcome variables were assigned using multiple imputation via chained equations. Values are mean ± standard deviation unless otherwise indicated. P-values presented are for the difference in change between baseline and 3 or 6 month assessments for the two treatment groups. The results presented are for adjusted analyses. CPAP=continuous positive airway pressure; FSMC=Fatigue Scale for Motor and Cognitive Functions; PSQI=Pittsburgh Sleep Quality Index; ESS=Epworth Sleepiness Scale; EDSS=Expanded Disability Status Scale;

VAS=visual analog scale; CES-D=Centers for Epidemiological Studies-Depression Scale; MSQOL-54=Multiple Sclerosis Quality of Life-54; MoCA=Montreal Cognitive Assessment.

Table S5. Blinding

	Active CPAP group-%	Sham CPAP group-%
Patients who guessed they were on active treatment	70 (12/17)	31 (5/16)
Patients that investigator guessed were on active treatment	41 (7/17)	19 (3/16)

Table S6. Changes in Outcome Measures According the Treatment Group at Six Months in Compliant Patients.

	Randomized to Fixed CPAP (n=11)		Randomized to Sham CPAP (n=5)		P-Values
	**Baseline**	**6 Months**	**Baseline**	**6 Months**	
FSS	6.5 ± 0.6	5.7 ± 1.0	6.1± 1.0	5.6 ± 1.2	0.59
FSMC					
Total score	78.0 ± 13.1	71.6 ± 18.6	77.0 ± 12.9	77.7 ± 18.4	0.35
Cognitive score	37.4 ± 7.8	34.0 ± 10.7	37.4 ± 5.2	37.0 ± 10.6	0.72
Motor score	40.6 ± 6.7	37.6 ± 9.1	39.6 ± 8.5	40.7 ± 9.8	0.23
Morning fatigue (proportion)	0.82	0.36	0.80	0.60	0.14
PSQI	8.7 ± 2.9	6.8 ± 2.3	12.2 ± 3.7	10.7 ± 2.2	0.84
ESS	11.9 ± 4.3	10.5 ± 5.2	11.0 ± 4.5	10.7 ± 5.5	0.98
EDSS	3.7 ± 2.0	3.8 ± 1.6	4.0 ± 2.1	3.8 ± 1.4	0.90
Pain due to illness (VAS)	35.3 ± 22.6	27.4 ± 25.8	35.6 ± 27.9	46.3 ± 25.1	0.74
Night pain (VAS)	31.7 ±30.5	26.8 ±19.0	32.4 ± 21.5	37.7 ± 19.6	0.79
CES-D	21.8 ± 5.9	19.5 ± 8.1	21.8 ± 5.1	18.7 ± 8.7	0.43
MSQOL-54					
Physical Composite Score	42.6 ± 10.5	45.6 ± 9.7	39.4 ± 16.6	34.7 ± 9.2	0.89
Mental Composite Score	35.0 ± 7.5	35.3 ± 10.0	33.9 ± 6.1	39.3 ± 10.7	**0.02**
MoCA	28.2 ± 1.6	27.3 ± 2.6	29.2 ± 1.3	29.3 ± 2.2	0.15
**Tower of London**					
Total correct score	5.0 ± 1.9	6.4 ± 2.6	6.0 ± 1.9	7.7 ± 2.5	0.68
Standard correct score	104.9 ± 12.0	113.5 ± 15.4	118.0 ± 19.0	128.7 ± 15.7	0.58
Total move score	28.3 ± 9.1	17.9 ± 13.2	17.0 ± 13.2	13.3 ± 17.1	0.44
Standard move score	102.2 ± 6.4	108.3 ± 14.5	115.6 ± 13.9	118.7 ± 14.1	0.78
Total time (mins)	314.6 ± 104.8	235.6 ± 72.2	300.0 ± 72.5	337.0 ± 72.4	0.12
Standard total time	95.6 ± 10.8	104.0 ± 6.7	99.2 ± 12.5	94.0 ± 6.9	0.19

Legend: Values are mean ± standard deviation unless otherwise indicated. P-values presented are for the difference in change between baseline and 6 month assessment for the two treatment groups. CPAP=continuous positive airway pressure; FSMC=Fatigue Scale for Motor and Cognitive Functions; PSQI=Pittsburgh Sleep Quality Index; ESS=Epworth Sleepiness Scale; EDSS=Expanded Disability Status Scale; VAS=visual analog scale; CES-D=Centers for Epidemiological Studies-Depression Scale; MSQOL-54=Multiple Sclerosis Quality of Life-54; MoCA=Montreal Cognitive Assessment

Table S7. Linear Regression Models of Association of MDAp with FSS and Change in FSS, Using Total Sleep Time (TST) from PSG and Using Sleep Time from PSQI.

**MDAp:**		**FSS**				**Change in FSS**			
		**Estimate**	**Std. Error**	**t value**	**Pr(>|t|)**	**Estimate**	**Std. Error**	**t value**	**Pr(>|t|)**
**Month 3**									
**Using TST**	**(Intercept)**	5.399	0.245	22.045	0.000	0.325	0.245	1.326	0.196
	**MDA**	-0.005	0.006	-0.864	0.395	0.002	0.006	0.372	0.713
**Using PSQI**	**(Intercept)**	5.392	0.248	21.768	0.000	0.332	0.244	1.363	0.183
	**MDA**	-0.001	0.007	-0.115	0.910	-0.001	0.007	-0.073	0.942
**Month 6**									
**Using TST**	**(Intercept)**	5.402	0.221	24.469	0.000	0.318	0.187	1.696	0.101
	**MDA**	-0.003	0.004	-0.648	0.523	0.000	0.004	-0.067	0.947
**Using PSQI**	**(Intercept)**	5.400	0.218	24.756	0.000	0.314	0.184	1.707	0.098
	**MDA**	0.000	0.005	-0.016	0.987	0.001	0.004	0.195	0.847

Legend: The first half of the table (FSS) shows results of linear regression analysis of association of MDAp on FSS at 3 and 6 months. The analysis was done using TST to calculate MDAp and again redone using PSQI to calculate the MDAp. The second part of the table shows the effect of MDAp on change in FSS at 3 and 6 months. Abbreviations: TST=Total sleep time; MDA=Mean disease alleviation; PSQI=Pittsburgh sleep quality index.
